# Network Analysis of Physical Activity, Personality, Goal Orientation and Depressive Subdimensions in College Students: Behavioral–Personality–Motivation Profiles

**DOI:** 10.3390/bs16071206

**Published:** 2026-07-17

**Authors:** Kangli Du, Qinshuo Zhang, Wenxue Ma, Yuyang Nie, Tianci Wang, Wenfeng Tan, Jiashuo Qi, Zhenzhuo Wang, Hongcheng Cui, Aihua Li, Cong Liu

**Affiliations:** 1 College of Education for the Future, Beijing Normal University, Zhuhai 519087, China; 2 College of Physical Education and Sports, Beijing Normal University, Beijing 100875, China; 3 School of Physical Education, Ningxia University, Yinchuan 750021, China; 4 College of Resources and Environment, Shandong Agricultural University, Taian 271018, China; 5 Leisure and Digital Sports College, Guangzhou Sports University, Guangzhou 510500, China; 11473@gzsport.edu.cn

**Keywords:** college students, depression symptoms, physical activity, Big Five personality traits, goal orientation, network analysis, latent profile analysis

## Abstract

Depressive symptoms are prevalent among college students. This study examined cross-domain associations among depressive symptom dimensions, physical activity, Big Five personality traits, and goal orientation using network analysis, latent profile analysis (LPA), multi-group network analysis, and multiple linear regression. LPA was conducted using behavioral, personality, and motivational variables only, with depressive symptom indicators excluded from class extraction. Data from 911 Chinese undergraduates were analyzed. The results showed that psychological equivalents, a cognitive-related depressive subdimension, had the highest strength centrality in the overall network, while neuroticism showed prominent positive bridge expected influence between personality traits and depression-related indicators. In the regression models, task orientation, agreeableness, conscientiousness, and openness were negatively associated with depressive symptoms to varying degrees. MVPA and sedentary time were not significantly associated with depressive symptoms in the total-sample model, whereas walking showed a subgroup-specific negative association only in the maladaptive, low-motivation, inactive profile. Three behavioral–personality–motivation profiles were identified, and the maladaptive profile showed relatively higher depressive symptom levels and stronger neuroticism-related associations. These findings provide associative, network- and profile-based evidence for understanding depressive symptoms among college students and may inform future longitudinal and intervention studies targeting behavioral–personality–motivation heterogeneity.

## 1. Introduction

Against the backdrop of rapid socioeconomic development and the popularization of higher education in China, college students are confronted with multiple stressors including academic competition, interpersonal adaptation, and career planning. Depressive symptoms have emerged as a prominent public health concern, severely threatening their physical and mental well-being as well as long-term development (Jiang et al., 2025). According to the World Health Organization (WHO) report in 2022, nearly 1 billion people worldwide suffer from mental health disorders (World Health Organization, 2022). Beyond persistent low mood and cognitive impairment, depression among college students is closely associated with reduced academic motivation, social withdrawal, and in severe cases, life-threatening behaviors—imposing substantial burdens on individual growth and social stability (Peng et al., 2021). From a developmental perspective, the university years constitute a critical period for personality maturation and identity formation. During this stage, individuals are exposed to intensive stress while their psychological resilience remains relatively underdeveloped, rendering them highly vulnerable to depressive symptoms. Consequently, an in-depth exploration of the influencing factors and underlying mechanisms of depression in this population, alongside the construction of scientific prevention and intervention systems, has become an urgent priority in the field of mental health research.

Physical activity, as a low-cost and easily implementable health promotion strategy, has garnered extensive scholarly attention regarding its association with mental health (Nie et al., 2025b). Accumulating evidence indicates that moderate-to-vigorous physical activity (MVPA) can effectively alleviate depressive symptoms through physiological pathways such as regulating cerebral neurotransmitter secretion and improving endocrine balance (Y. Wang & Li, 2022; Zhu et al., 2023). In contrast, prolonged sedentary behavior may increase depression risk by reducing social interaction and impairing metabolic efficiency (Zhou et al., 2021). However, the effects of different types of physical activity on depression vary substantially: some studies have failed to detect significant correlations between walking, sedentary time, and depression (Pengpid & Peltzer, 2019), suggesting that the relationship between physical activity and depression is not simply linear. Recent studies have further shown that this field is rapidly moving beyond simple direct associations. For example, sports activities have been linked to depression and anxiety through the mediating role of self-esteem (Zhao et al., 2025), while physical activity has also been found to shape students’ academic motivation through mental health (D. Ma et al., 2026). In addition, exercise participation has been associated with depressive symptoms through behavioral inhibition and activation systems among college students (Li et al., 2023). This complex association may be moderated by other variables such as personality traits and cognitive patterns, and its specific pathways remain to be further clarified.

Personality traits, as stable psychological and behavioral characteristics of individuals, are core psychological predictors of the onset and progression of depression. The Big Five personality model comprises five core traits: extraversion, agreeableness, conscientiousness, neuroticism, and openness. Cumulative research consistently identifies neuroticism (emotional instability) as a robust positive correlate of depression. Those with elevated neuroticism are vulnerable to sustained negative emotional spirals, driven by greater emotional sensitivity and weaker psychological resilience against stress (Miscioscia et al., 2022; W. Ma et al., 2025). Conversely, positive personality traits such as agreeableness, conscientiousness, and openness exert protective effects against depression by enhancing interpersonal adaptability and self-regulation capacity (Deng et al., 2020). Recent evidence has also suggested that Big Five personality traits may influence exercise motivation and mental health through emotional intelligence, highlighting the importance of considering personality-related mechanisms in the physical activity–mental health relationship (Y. Zhang, 2025). Additionally, goal orientation theory posits that individuals’ goal orientations (self-oriented vs. task-oriented) significantly influence their attitudes and behaviors when coping with setbacks (W. Wang et al., 2021). Task-oriented individuals focus more on personal ability improvement and demonstrate greater psychological resilience in the face of difficulties, whereas self-oriented individuals tend to compare themselves with others, making them vulnerable to self-denial and negative emotions following failures (Duda & Nicholls, 1992). At the behavioral–motivational level, physical activity enjoyment, exercise self-efficacy, physical activity recording, and exercise goal setting have been shown to be associated with college students’ physical activity levels (Taylor et al., 2025), suggesting that motivational processes may be closely intertwined with activity participation and psychological adaptation. Nevertheless, research on the direct association between goal orientation and depression, as well as its interactive effects with personality traits and physical activity, remains limited, and consistent conclusions have not been reached.

This study is grounded in a multidimensional integrated theoretical framework that systematically links physical activity, Big Five personality traits, achievement goal orientation, and depressive symptoms. First, Big Five personality theory provides the core basis for understanding personality vulnerability to depression, with neuroticism as a transdiagnostic risk factor and other traits as protective factors. Second, achievement goal orientation theory explains how motivational patterns shape emotional adaptation and resilience. Third, the psychobiological model of physical activity and mental health highlights the intensity-specific benefits of MVPA compared with walking or sedentary time.

Previous studies have predominantly employed variable-centered approaches, such as traditional regression analysis, which focus on the direct predictive effects of individual variables on depression. However, this analytical strategy falls short of capturing the complex interactive networks among multidimensional factors, including physical activity, the Big Five personality traits, and goal orientation (Dong et al., 2024). As an emerging relation-centered methodology, network analysis offers an intuitive representation of the interconnections among variables, identifies core hub nodes, and reveals cross-dimensional pathways, thereby offering a novel lens for understanding the multifactorial mechanisms underlying depression (M. Zhang et al., 2024; Cheng et al., 2026). In parallel, latent profile analysis enables the identification of person-centered subgroups based on patterns of physical activity, personality, and goal orientation, thus overcoming the limitation of aggregate models that overlook population heterogeneity (Prochnow et al., 2020; Liu et al., 2026). The integration of network analysis and latent profile analysis holds the potential to address two critical questions: which variables exert the most pivotal influence, and which student subgroups may require tailored intervention strategies.

To address these research gaps, the present study integrates the above theoretical framework and adopts a combined method of network analysis and multiple linear regression analysis with undergraduate students from comprehensive universities as participants, aiming to explore the network structure characteristics and core hub nodes among physical activity, personality traits, goal orientation, and depression-related indicators, examine the direct predictive effects and relative importance of variables in each dimension on depression, and identify heterogeneous behavioral–personality–motivation subgroups with distinct depressive symptom correlation patterns.

Based on the integrated theoretical framework and prior empirical evidence, the following a priori, testable hypotheses were formulated:

**H1.**  *Neuroticism will act as the primary bridge node connecting personality traits to depression, while agreeableness, conscientiousness, and openness will show significant negative associations with depression.*

**H2.**  *Task-orientation will serve as a protective factor and negatively predict depression, whereas self-orientation will show weak or non-significant associations.*

**H3.**  *MVPA will be negatively associated with depression, while walking and sedentary time will show weak or non-significant links with depression-related indicators.*

**H4.**  *The network structures of study variables are expected to show exploratory descriptive heterogeneity across behavioral–personality–motivation latent subtypes, with potentially distinct variable association patterns across subgroups.*

This study aims to examine multidimensional associations among physical activity, personality traits, goal orientation, and depressive symptoms in college students, thereby providing exploratory evidence that may inform future longitudinal and intervention studies considering behavioral–personality–motivation heterogeneity.

## 2. Method

### 2.1. Participants

This cross-sectional study adopted convenience sampling for participant recruitment. Survey respondents were full-time undergraduate students spanning all four academic years (freshmen to seniors) enrolled in comprehensive universities across mainland China. Data collection was conducted from 1 to 31 July 2025, through the Wenjuanxing platform, which features multiple quality control mechanisms. Mandatory response logic was implemented throughout the survey, and all items included two attention-check questions that required complete responses before submission, thereby reducing omissions and mid-survey abandonment. A total of 1025 questionnaires were distributed. After manual verification and algorithmic screening (duplicate IP identification and response duration threshold filtering), 1023 responses were initially retained. Subsequently, 112 invalid questionnaires were excluded for failing attention-check questions or having response durations exceeding ±3 standard deviations, yielding a final sample of 911 valid questionnaires with an effective response rate of 88.9%. The sample comprised 236 males (25.9%) and 675 females (74.1%). Detailed demographic characteristics including age, major, and grade level are presented in Table 1. All procedures strictly adhered to the Declaration of Helsinki and the Chinese Psychological Society’s ethical guidelines and were approved by the university’s Institutional Review Board (Approval No.: BNU202506160168).

### 2.2. Instruments

#### 2.2.1. Physical Activity

Physical activity was measured via International Physical Activity Questionnaire-Short Form (IPAQ-SF). This tool captures the weekly frequency (days/week) and daily duration (min/day) of walking, moderate-intensity, and vigorous-intensity activities, in addition to sedentary behavior (Craig et al., 2003). Energy expenditure was calculated as Metabolic Equivalent of Task (MET)-minutes per week, using established MET coefficients: 3.3 for walking, 4.0 for moderate-intensity activity, and 8.0 for vigorous-intensity activity (Ainsworth et al., 2011). On the basis of established IPAQ-SF scoring protocols (IPAQ Research Committee, 2005), participants were categorized into low, moderate, or high physical activity levels. In the present study, moderate-to-vigorous physical activity (MVPA) was operationalized as the combined MET-minutes per week from moderate- and vigorous-intensity activities. Because the IPAQ-SF assesses heterogeneous behavioral components rather than a homogeneous latent psychological construct, internal consistency reliability and confirmatory factor analysis were not calculated for this measure.

#### 2.2.2. Depressive Symptoms

Depressive symptoms were assessed using the Self-Rating Depression Scale (SDS) (Zung, 1965). This 20-item self-report instrument employs a 4-point Likert scale to evaluate three core dimensions: pervasive affect, physiological equivalents, psychological equivalents. The SDS is widely employed for preliminary screening, monitoring treatment response, and related scientific research. In the present sample, the SDS demonstrated good internal consistency for the total scale (Cronbach’s α = 0.838). The internal consistency coefficients for the three symptom domains were α = 0.738 for pervasive affect, α = 0.693 for physiological equivalents, and α = 0.715 for psychological equivalents. Confirmatory factor analysis of the three-factor model did not show satisfactory fit in the present sample, χ^2^/df = 8.506, CFI = 0.769, TLI = 0.738, RMSEA = 0.075, and SRMR = 0.096. Therefore, the SDS total score was used as the primary indicator of overall depressive symptom burden in the regression analyses, whereas the three symptom domains were included only in the network analysis to preserve the multidimensional structure of depressive symptoms. Given the limited support for the three-factor structure in this sample, findings involving SDS subdimensions were interpreted cautiously.

#### 2.2.3. Goal Orientation

Goal orientations were assessed using the 13-item TEOSQ (Dagsdóttir et al., 2023), originally developed for sport settings, including task and self-orientation subscales with 5-point Likert scoring. The item stem/instruction was adapted to refer to general academic and campus-life contexts, whereas the item content was otherwise retained. Goal orientation is a cross-situational dispositional trait, and prior work supports TEOSQ’s validity for non-athletic undergraduates. In this sample, the overall Cronbach’s α of TEOSQ reached 0.948; subscale reliability coefficients were α = 0.924 for task orientation and α = 0.905 for self-orientation. Confirmatory factor analysis of the two-factor model showed acceptable fit according to CFI, TLI, and SRMR, although RMSEA was higher than the conventional cutoff, χ^2^/df = 10.948, CFI = 0.930, TLI = 0.914, RMSEA = 0.105, and SRMR = 0.047. Thus, the TEOSQ showed partially acceptable structural validity in the present sample, and the two subscale scores were used in subsequent analyses with caution.

#### 2.2.4. Big Five Personality Traits

Big Five personality traits were assessed using the Big Five Inventory-2 Short Form (BFI-2-SF) (Soto & John, 2017). The BFI-2-SF is a 15-item measure developed to assess five broad personality traits, extraversion, agreeableness, conscientiousness, neuroticism, and openness to experience, with three items for each dimension. Reverse-scored items were recoded before calculating dimension scores. In the present sample, internal consistency coefficients for the five BFI-2-SF dimensions were relatively low: α = 0.622 for extraversion, α = 0.490 for agreeableness, α = 0.500 for conscientiousness, α = 0.663 for neuroticism, and α = 0.528 for openness. Confirmatory factor analysis of the five-factor correlated model also showed unsatisfactory fit, χ^2^/df = 8.921, CFI = 0.742, TLI = 0.661, RMSEA = 0.081, and SRMR = 0.074. Given the limited internal consistency and structural validity of the BFI-2-SF in the present sample, personality traits were retained in subsequent analyses only as exploratory or control variables. Therefore, findings involving personality variables in the network structure, latent profile labels, and regression models should be interpreted cautiously and should not be regarded as confirmatory evidence of stable personality mechanisms.

### 2.3. Data Analysis

Data analysis integrated network analysis, latent profile analysis (LPA), and multiple regression, implemented in RStudio (2025.09.1+401) and Mplus 9 (R Core Team, 2016). Harman’s single-factor test assessed common method bias. Descriptive statistics (mean, standard deviation, skewness, kurtosis) were calculated for all variables. Because the three physical activity indicators were right-skewed, MVPA, walking, and sedentary time were transformed using log10(x + 1), yielding MVPA_Log, walking_Log, and sedentary_Log. Spearman rank correlation matrix was adopted as the input of EBICglasso estimation to address residual skewness of activity indicators, in accordance with standard non-paranormal transformation for skewed continuous data. Bivariate correlations and a heatmap illustrated variable associations. A Gaussian graphical model (GGM) was estimated using the extended Bayesian information criterion least absolute shrinkage and selection operator (EBICglasso) to examine conditional associations among 13 variables, including physical activity indicators, Big Five personality traits, goal orientation, and three depression-related dimensions (Meinshausen & Bühlmann, 2006; Foygel & Drton, 2010). Node centrality, expected influence, and bridge metrics identified key nodes and cross-domain pathways (Epskamp et al., 2018; Jones et al., 2021). Network stability was validated via case-dropping subset bootstrap and non-parametric bootstrap. Latent profile analysis (LPA) was conducted in Mplus using ten behavioral, personality, and motivational indicators (Collins & Lanza, 2013). Before LPA, the three skewed physical activity variables were transformed using log10(x + 1) and entered as MVPA_Log, walking_Log, and sedentary_Log. The remaining seven indicators were extraversion, agreeableness, conscientiousness, neuroticism, openness, self-orientation, and task-orientation, which were entered using their original scale-score metrics. Depressive symptom indicators were not included in class extraction. The three-class solution was selected by considering model fit indices, entropy, class size, parsimony, and substantive interpretability. Multiple linear regression models were then conducted for the total sample and each latent profile. In these models, the SDS total score was used as the dependent variable to represent overall depressive symptom burden, and physical activity indicators, Big Five personality traits, and goal orientation variables were entered simultaneously as predictors. Adjusted R^2^, F-values, standardized coefficients, significance levels, and regression diagnostics were reported (Cohen et al., 2013).

## 3. Results

### 3.1. Common Method Bias Test

A Harman’s single-factor test (Tang & Wen, 2020) revealed that the first unrotated factor accounted for 22.55% of the total variance, which is below the 40% threshold. This indicates that common method bias was not a significant concern in this study.

### 3.2. Preliminary Descriptive Analyses

With a valid sample of 911 and no missing data (see Table 2), raw MVPA, walking time and sedentary time showed severe right-skewed and leptokurtic distribution and violated normality, while psychological variables including Big Five personality, orientation, pervasive affect, psychological equivalents and depression total score approximately followed normal distribution and could be directly used for parametric analysis. The three log-transformed physical activity variables showed improved distributional properties; despite mild residual skewness and kurtosis, they were considered suitable for correlation, network, and regression analyses.

### 3.3. Correlation Analysis

**Figure 1:**
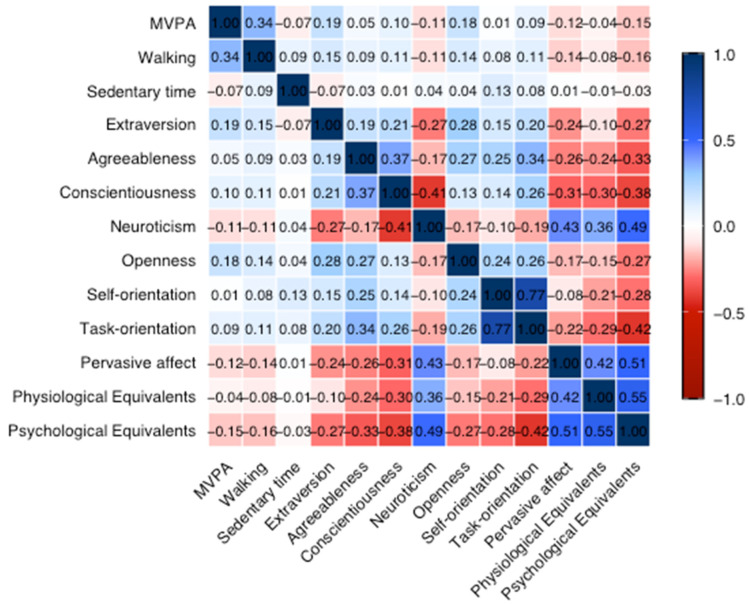
Partial Correlation Coefficients Between Nodes.

Figure 1 presents a correlation heatmap illustrating the bivariate associations among all study variables. Moderate-to-vigorous physical activity (MVPA) showed a moderate positive correlation with walking (r = 0.34), weak positive correlations with extraversion and task-orientation, and weak negative correlations with neuroticism and psychological equivalents. Conversely, walking and sedentary time exhibited negligible associations with most variables.

Within the personality domain, self-orientation was strongly positively correlated with task-orientation (r = 0.77), while conscientiousness showed moderate correlations with both agreeableness and neuroticism. Regarding psychological states, pervasive affect, physiological equivalents, and psychological equivalents formed a distinct cluster characterized by moderate-to-strong positive inter-correlations.

Overall, intra-dimensional associations were markedly stronger than cross-dimensional ones. Positive personality traits and MVPA were predominantly associated with positive psychological states, whereas inverse patterns were observed with negative traits, a pattern consistent with the “positive behavior-positive psychology” framework.

### 3.4. Network Analysis

#### 3.4.1. Network Structure

**Figure 2:**
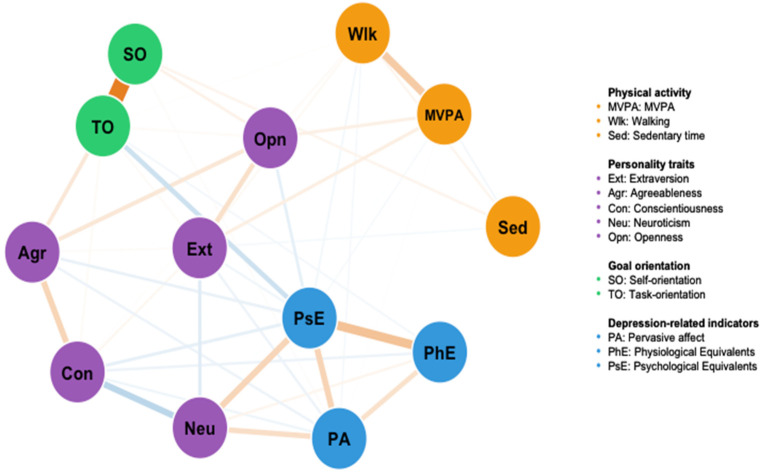
Network Structure of Physical Activity, Personality Traits, Orientation, and Depression-Related Indicators.

As shown in Figure 2, the estimated network included physical activity indicators, personality traits, orientation variables, and depression-related indicators. The depression-related nodes were mainly located in the lower-right area of the network, forming a tightly connected cluster. Psychological equivalents (PsE) showed the strongest connection with physiological equivalents (PhE), with additional strong links to pervasive affect (PA), indicating close associations among all depression-related dimensions. Personality trait nodes were distributed across the middle-left of the network. Neuroticism (Neu) showed a clear, moderate connection with PsE, while conscientiousness (Con) and agreeableness (Agr) also had a notable link to PsE, suggesting important associations between these personality traits and depression-related indicators. Within the goal orientation community, self-orientation (SO) and task-orientation (TO) were strongly connected by the thickest edge in the entire network, highlighting a very close relationship between these two constructs. Physical activity nodes were located in the upper-right part of the network. Walking (Wlk) and MVPA showed a moderate positive connection, while sedentary time (Sed) was weakly connected to the other two activity indicators, reflecting consistency between active physical activity indicators.

#### 3.4.2. Centrality Indices

**Figure 3:**
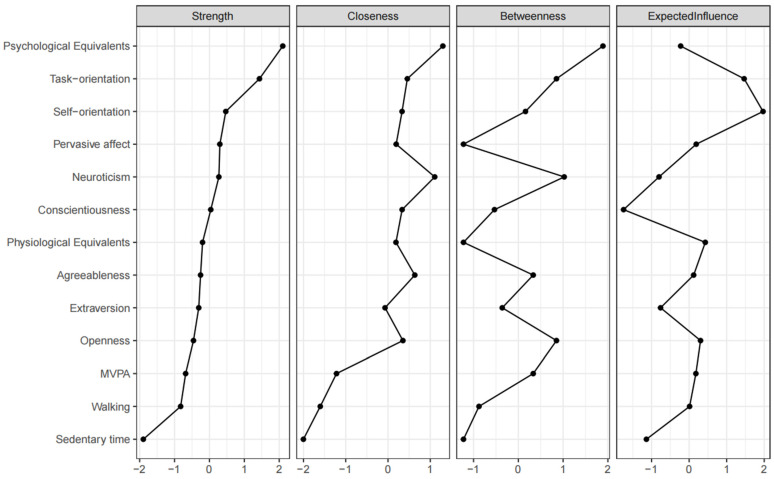
Comparison of nodes on strength, closeness, betweenness, and expected influence.

The centrality results are presented in Figure 3 and Table 3. Among the depressive symptom dimensions, psychological equivalents (PsE) emerged as the most central node in the network, exhibiting the highest strength (Z = 2.10) and betweenness (Z = 1.89) centrality. Task orientation (Z = 1.43) and self-orientation (Z = 0.47) also showed relatively high strength, reflecting their interconnections with other variables. Betweenness centrality further highlighted secondary bridging roles for openness and task-orientation (both Z = 0.85). In terms of expected influence, self-orientation (Z = 1.97) and task-orientation (Z = 1.47) demonstrated the highest values, suggesting that goal orientation variables exerted the strongest overall influence in the network. Conversely, sedentary time showed the lowest values across key centrality indices, confirming its peripheral position.

#### 3.4.3. Bridge Centrality Indices

**Figure 4:**
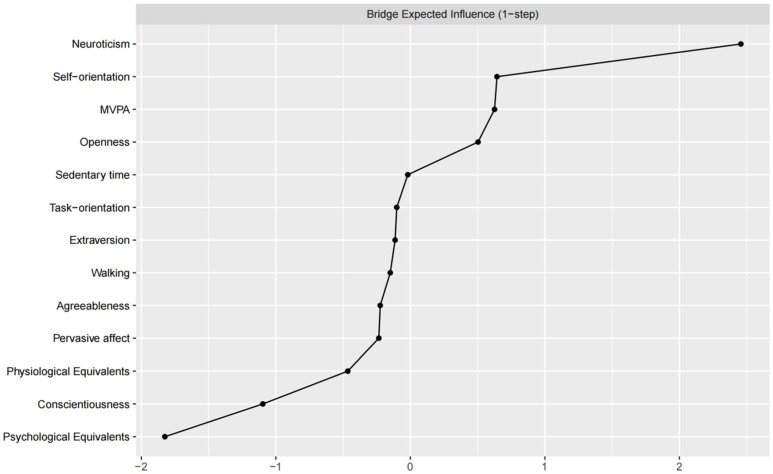
Bridge Expected Influence analysis.

The bridge centrality results further revealed the roles of variables in connecting distinct communities. As shown in Figure 4, neuroticism exhibited the highest positive bridge expected influence, followed by self-orientation and MVPA, indicating that these variables act as key positive bridges linking different domains. In contrast, psychological equivalents (PsE) showed the strongest negative bridge expected influence, followed by conscientiousness, indicating that its cross-community connections were predominantly negative in direction. Given the focus on depression-related symptoms, negative bridge expected influence reflects protective patterns, whereby higher levels of adaptive traits or behaviors are associated with lower depressive symptoms.

**Figure 5:**
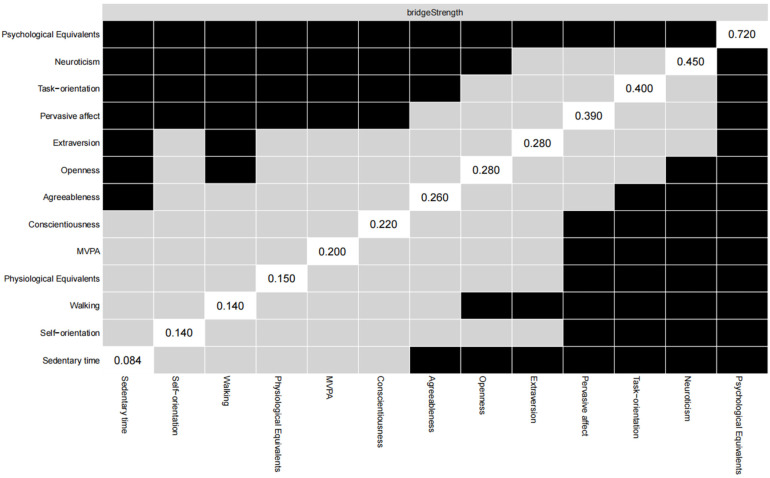
Bridge Strength matrix. Black cells indicate non-significant connections; gray cells show significant bridge strength with numeric labels.

Complementing these findings, Figure 5 shows that PsE had the highest bridge strength (0.72), indicating the strongest overall cross-community connectivity, followed by neuroticism (0.45), task-orientation (0.40), and pervasive affect (0.39). It should be noted that bridge strength reflects only the magnitude of cross-community connections, whereas bridge expected influence retains their direction. Thus, although PsE was the most prominent cross-community connector in terms of strength, its bridging role was characterized by negative associations. Conversely, neuroticism showed both high bridge strength and the highest positive bridge expected influence, suggesting it functions as the most critical positive risk-related bridge node in the network.

#### 3.4.4. Stability Analysis

**Figure 6:**
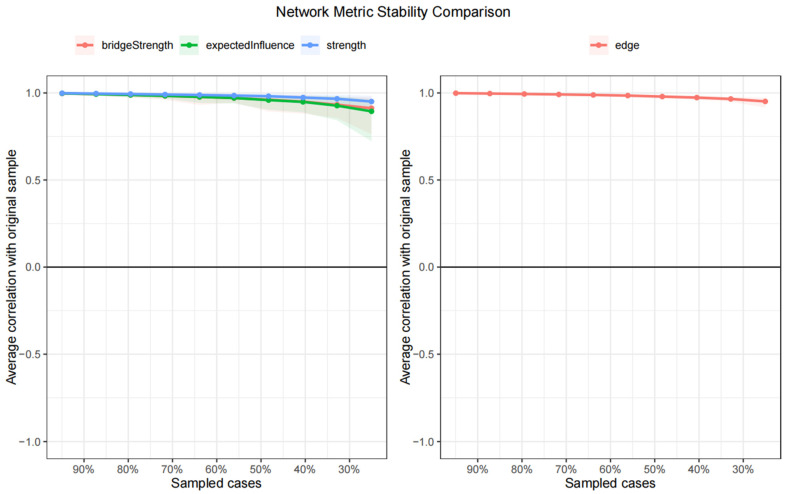
Correlation Stability of Network Metrics with Sample Size.

Stability was evaluated using the case-dropping bootstrap procedure. As shown in Figure 6, the correlation-stability coefficients (CS) for the core indices of the network were above the recommended threshold of 0.50: edge strength, strength centrality, bridge strength, and expected influence all showed CS values of 0.75, indicating excellent stability. Even after deleting up to 70% of cases, the mean correlations between the subsample estimates and the full-sample estimates remained high. These results suggest that the network structure, centrality indices, and bridge centrality findings were highly robust to sample reduction, supporting the reliability of the core findings.

### 3.5. Latent Profile Analysis

**Figure 7:**
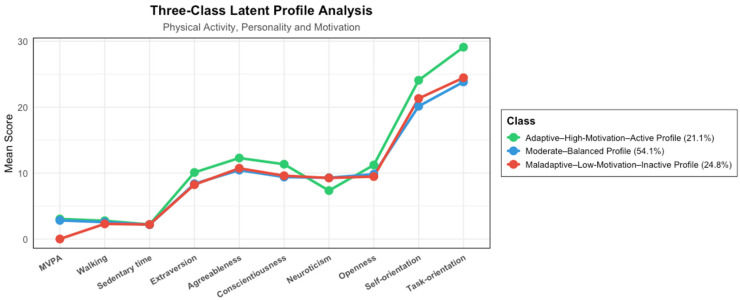
Mplus-estimated class-specific mean profiles for the three-class latent profile solution. Color grouping for each latent class is detailed in the embedded figure legend.

Latent profile analysis was conducted using ten indicators to identify latent heterogeneity among participants. Specifically, MVPA, walking, and sedentary time were log-transformed using log10(x + 1) and entered as MVPA_Log, walking_Log, and sedentary_Log. Extraversion, agreeableness, conscientiousness, neuroticism, openness, self-orientation, and task-orientation were entered using their original scale-score metrics. No z-score standardization was applied before LPA or profile plotting. Therefore, the profile values reported in Figure 7 represent Mplus-estimated class-specific means in the analyzed metric of each indicator rather than z-scores. Models with one to six classes were estimated to determine the optimal solution. The results showed that loglikelihood increased and AIC, BIC, and sample-size-adjusted BIC generally decreased as the number of classes increased, indicating improved model fit (see Table 4). However, the likelihood-ratio comparisons showed that the two-class and three-class models significantly improved fit over the preceding models, both *p* < 0.001, whereas the four-, five-, and six-class models did not provide significant additional improvement, with *p* = 0.6715, *p* = 0.056, and *p* = 0.4136, respectively. The entropy value of the three-class model was 0.858, indicating good classification accuracy. Considering model fit, classification quality, parsimony, and theoretical interpretability, the three-class solution was selected as the optimal model, suggesting that participants could be classified into three latent profiles with distinct patterns of physical activity, sedentary behavior, personality traits, and goal orientation.

As shown in Figure 7, the three-class latent profile model identified three distinct behavioral–personality–motivation profiles based on the Mplus-estimated class-specific means of the ten LPA indicators. Figure 7 displays mean scores in the analyzed metric of each indicator rather than standardized z-scores. Specifically, the physical activity indicators are shown as log-transformed values, whereas personality traits and goal-orientation variables are shown in their original scale-score metrics. Class 1 was labeled as the Adaptive-High-Motivation-Active Profile and included 192 participants, accounting for 21.1% of the sample. This class showed relatively higher levels of MVPA, walking, positive personality traits, self-orientation, and task-orientation, together with relatively lower neuroticism, reflecting a more adaptive behavioral–psychological configuration. Class 2 was labeled as the Moderate–Balanced Profile and included 493 participants, accounting for 54.1% of the sample. As the largest subgroup, this class showed generally average levels across physical activity, sedentary behavior, personality traits, and goal orientation. Class 3 was labeled as the Maladaptive–Low-Motivation–Inactive Profile and included 226 participants, accounting for 24.8% of the sample. This class was characterized by relatively lower MVPA, walking, positive personality traits, and goal orientation but relatively higher neuroticism, indicating a less favorable behavioral–personality–motivation configuration. Because depressive symptom indicators were not used to derive the profiles, these classes should be interpreted as behavioral–personality–motivation profiles rather than depression-severity groups. Moreover, given the psychometric limitations of the BFI-2-SF in this sample, the profile labels are descriptive and exploratory. They should not be interpreted as stable personality-based typologies, but rather as sample-specific configurations based on the observed behavioral, personality, and motivational indicators.

### 3.6. Network Heterogeneity Across Latent Profiles

**Figure 8:**
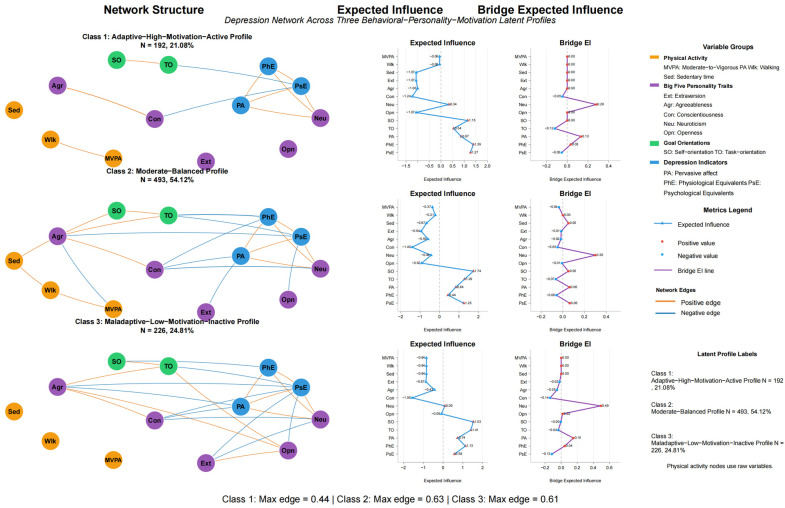
Network structure, expected influence, and bridge expected influence across three behavioral–personality–motivation latent profiles. Detailed color and symbol interpretations are provided in the embedded figure legend.

The following cross-profile network comparisons are purely descriptive and exploratory, as formal statistical invariance tests were not conducted. The maximum edge weights were 0.44 for Class 1, 0.63 for Class 2, and 0.61 for Class 3 (see Figure 8), indicating that the strongest pairwise conditional association was smaller in Class 1 than in Class 2 and Class 3. These values suggest stronger local associations in the moderate-balanced and maladaptive-low-motivation-inactive profiles but should be interpreted only as descriptive patterns rather than statistically tested between-profile differences.

In Class 1, the Adaptive-High-Motivation-Active Profile, physiological equivalents and psychological equivalents showed the highest expected Influence values, PhE = 1.39 and PsE = 1.27, followed by self-orientation, SO = 1.15, pervasive affect, PA = 0.87, and task-orientation, TO = 0.54. The strongest negative expected Influence value was observed for conscientiousness, Con = −1.24. Several other nodes also showed negative expected Influence values, including sedentary time, extraversion, openness, agreeableness, walking, and MVPA. For bridge expected influence, neuroticism was the strongest positive bridge node, Neu = 0.28, followed by pervasive affect, PA = 0.13, whereas task-orientation showed a negative bridge value, TO = −0.12.

In Class 2, the Moderate-Balanced Profile, self-orientation and task-orientation showed the highest expected Influence values, SO = 1.74 and TO = 1.29, followed by psychological equivalents, PsE = 1.25, pervasive affect, PA = 0.84, and physiological equivalents, PhE = 0.44. The strongest negative expected Influence values were found for conscientiousness, Con = −1.40, extraversion, Ext = −0.94, openness, Opn = −0.92, and sedentary time, Sed = −0.67. For bridge expected influence, Neuroticism again showed the highest positive value, Neu = 0.30, suggesting that it was the most prominent positive cross-community connector in this profile.

In Class 3, the Maladaptive-Low-Motivation-Inactive Profile, self-orientation and task-orientation remained the most influential nodes, SO = 1.53 and TO = 1.41, followed by physiological equivalents, PhE = 1.13, pervasive affect, PA = 0.74, and psychological equivalents, PsE = 0.58. Physical activity-related nodes showed consistently negative expected Influence values, including MVPA = −0.84, walking = −0.84, and sedentary time = −0.84, while conscientiousness showed the strongest negative value, Con = −1.56. For bridge expected influence, neuroticism had the highest positive value, Neu = 0.49, exceeding its corresponding values in Class 1 and Class 2, followed by pervasive affect, PA = 0.15. These descriptive patterns suggest that neuroticism may play a particularly important cross-domain bridging role in the maladaptive, low-motivation, inactive subgroup.

### 3.7. Multiple Linear Regression Analysis

Regression models were established for the total sample and three latent profiles to explore predictors of depressive symptoms. All regression models reached statistical significance, *p* < 0.001. The adjusted R^2^ was 0.389 in the total sample, 0.318 in Class 1, 0.197 in Class 2, and 0.348 in Class 3, indicating moderate explanatory power in the total sample and Class 3, and relatively lower explanatory power in Class 2 (see Table 5).

Regression diagnostics indicated no severe multicollinearity. VIF values ranged from 1.070 to 3.372 in the total sample, from 1.083 to 1.952 in Class 1, from 1.038 to 3.029 in Class 2, and from 1.054 to 3.589 in Class 3. All tolerance values were above 0.279. Although the condition indices suggested shared variance between self-orientation and task-orientation, the VIF values remained within acceptable limits. Residual diagnostics based on standardized residuals, residual-versus-predicted plots, and normal probability plots did not indicate serious violations of regression assumptions.

In the total sample, neuroticism was positively associated with depressive symptoms, whereas agreeableness, conscientiousness, openness, and task orientation were negatively associated with depressive symptoms. Physical activity indicators and sedentary time showed no significant associations with depressive symptoms in the total-sample model.

Subgroup analyses revealed both consistent and heterogeneous patterns across latent profiles. Neuroticism positively predicted depressive symptoms in all three classes and showed the strongest standardized coefficient in Class 3. Agreeableness was negatively associated with depressive symptoms in Class 1 and Class 2, but not in Class 3. Conscientiousness was significant in Class 1 and Class 3, whereas openness was significant only in Class 2. Task orientation was negatively associated with depressive symptoms in Class 1 and Class 2, but not in Class 3. Self-orientation showed a small positive association only in Class 2. Among physical activity indicators, walking was negatively associated with depressive symptoms only in Class 3, whereas MVPA and sedentary time were non-significant across all subgroup models.

## 4. Discussion

This study examined the associations among depressive symptoms, physical activity, personality traits, and goal orientation in college students by integrating network analysis, latent profile analysis, and multiple linear regression. Overall, the findings suggest that depressive symptoms were statistically connected with personality, motivational, and behavioral factors within a multidimensional association network. In the overall network, neuroticism showed prominent positive bridge expected influence between personality traits and depression-related indicators, while task orientation, agreeableness, conscientiousness, and openness were negatively associated with depressive symptoms in the regression models to varying degrees. Physical activity indicators showed limited direct associations with depressive symptoms in the overall regression model, although a subgroup-specific negative association between walking and depressive symptoms was observed in the maladaptive, low-motivation, inactive profile. However, given the cross-sectional design, these findings should be interpreted as associative rather than causal or mechanistic evidence.

First, the network analysis revealed a closely connected cluster of depression-related dimensions, particularly the strong association between psychological equivalents and physiological equivalents. This finding suggests that depressive symptoms among college students may involve interrelated cognitive–psychological and somatic domains, rather than being limited to affective disturbances alone. Psychological equivalents showed the highest strength centrality, indicating that this depressive subdimension had the strongest overall statistical connections with other nodes in the estimated cross-sectional network. This pattern is broadly consistent with X. H. Zhang et al. (2026), who reported that depressive symptoms occupied a central position in a multilayer network analysis of UK university students (X. H. Zhang et al., 2026). Taken together, these findings suggest that cognitive-related depressive experiences, such as negative self-evaluation and related psychological complaints, may represent an important domain for future longitudinal and intervention research.

Second, the latent profile analysis further revealed behavioral–personality–motivation heterogeneity within the college student sample. Because the profiles were derived from physical activity, personality traits, and goal orientation variables rather than depressive symptom indicators, they should be understood as behavioral–psychological configurations rather than depression-severity groups. In brief, the maladaptive, low-motivation, inactive profile showed relatively higher depressive symptom levels and stronger neuroticism-related associations, whereas the adaptive, high-motivation, active profile showed a more favorable behavioral–psychological configuration. These findings suggest that depressive symptoms may co-occur with broader patterns of unfavorable behavioral, personality, and motivational characteristics rather than being associated with a single isolated factor (Borghuis et al., 2020; Woo et al., 2018). Similar profile-based evidence has shown that exercise participation and psychological resources may combine into distinct subgroups associated with academic stress, further supporting the value of person-centered approaches (Qu et al., 2026). Given the cross-sectional design, these profile-based differences should be interpreted as descriptive and associative patterns rather than evidence of causal developmental pathways.

Third, neuroticism showed the highest positive bridge expected influence in the bridge centrality analysis, suggesting that it may serve as a prominent cross-community correlate between the personality domain and depression-related indicators in this sample. This pattern is broadly consistent with previous evidence indicating that individuals with higher neuroticism tend to be more sensitive to stressors, more prone to negative affect (Mikkelsen et al., 2024), and more likely to show maladaptive response patterns, which may be associated with greater vulnerability to depressive symptoms. The regression results were also consistent with this pattern: neuroticism was positively associated with depressive symptoms in the total sample and across all three latent profiles, with the largest standardized coefficient observed in the maladaptive, low-motivation, inactive profile. These findings suggest that neuroticism may be relevant to understanding depressive symptom differences among college students, particularly within the maladaptive profile (Meiering et al., 2025). However, because the BFI-2-SF showed psychometric limitations in the present sample, this finding should be interpreted cautiously. Therefore, neuroticism should be viewed as an exploratory risk-related correlate rather than a confirmed psychological mechanism or direct intervention target. Future studies using longer and psychometrically stronger personality measures are needed to determine whether this pattern can be replicated.

Fourth, task orientation showed a relatively consistent negative association with depressive symptoms, although this pattern should be interpreted cautiously. In the overall network, task orientation showed relatively high strength and expected influence, indicating that it was closely connected with other nodes in the estimated cross-sectional association network. In the regression analyses, task orientation was significantly and negatively associated with depressive symptoms in the total sample, Class 1, and Class 2, but this association was not significant in Class 3. These results suggest that students who focus on personal improvement, effort, mastery, and self-referenced progress may be more likely to develop adaptive coping strategies and psychological resilience (Truninger et al., 2018; Qiu et al., 2025). In contrast, self-orientation was strongly connected with task orientation and showed high expected influence in the network, but it did not significantly predict depression in the regression models. This pattern suggests that the mental health implications of self-orientation may vary across motivational and personality contexts. Future longitudinal studies should further distinguish adaptive competitive motivation from maladaptive social comparison and examine how different goal-orientation patterns are associated with changes in depressive symptoms over time.

Fifth, agreeableness, conscientiousness, and openness showed varying degrees of negative associations with depressive symptoms across models. Agreeableness was negatively associated with depressive symptoms in the total sample, Class 1, and Class 2, but not in Class 3. Conscientiousness showed negative associations in the total sample, Class 1, and Class 3, whereas openness showed negative associations only in the total sample and Class 2. These results suggest that the associations between specific personality traits and depressive symptoms may differ across behavioral–personality–motivation profiles, rather than following a uniform pattern across all subgroups (X. Y. Wang et al., 2025; Aguirre et al., 2024). However, these personality-related findings should be interpreted cautiously. Given the cross-sectional design and the psychometric limitations of the BFI-2-SF dimensions in the present sample, the observed associations should be regarded as exploratory and sample-specific rather than evidence of stable protective personality mechanisms. Future longitudinal studies using longer and psychometrically stronger personality measures are needed to determine whether these profile-specific patterns can be replicated.

Sixth, the association between physical activity indicators and depressive symptoms appeared limited and subgroup-specific in the present study. Although moderate-to-vigorous physical activity has been widely discussed as beneficial for mental health (Chekroud et al., 2018), MVPA and sedentary time were not significantly associated with depressive symptoms in either the total-sample regression model or any latent-profile-specific model. This finding suggests that, in this sample, physical activity indicators did not show a broad direct association with depressive symptoms after accounting for personality traits and goal orientation. The role of physical activity may depend on factors such as activity type, activity context, behavioral consistency, motivation, and personality characteristics (Nie et al., 2025a; Lubans et al., 2016). Notably, walking was negatively associated with depressive symptoms only in the maladaptive, low-motivation, inactive profile. This subgroup-specific finding suggests that lower-threshold daily activity may be relevant to depressive symptoms among students with lower motivation and activity levels (Robertson et al., 2012). Future longitudinal or intervention studies using objective activity measures are needed to determine whether walking or other forms of physical activity can contribute to changes in depressive symptoms among high-risk student subgroups.

Several limitations should be acknowledged. First, the cross-sectional design limits causal and temporal interpretations of the associations among personality traits, goal orientation, physical activity, and depressive symptoms. Future studies should use longitudinal, cross-lagged, or ecological momentary assessment designs to examine dynamic changes among these variables. Second, this study used convenience sampling, which may limit sample representativeness and the generalizability of the findings. Future studies should adopt stratified, quota-based, or multi-center sampling strategies. Third, physical activity was assessed using self-report measures, which may be affected by recall bias and social desirability. Future research should incorporate accelerometers or wearable devices to obtain more objective activity indicators. Fourth, the sample showed a gender imbalance, with female students accounting for 74.1% of participants. Given potential gender differences in MVPA and depressive symptoms, this imbalance may have influenced the pooled estimates, network structure, latent profiles, and regression associations. Moreover, gender-stratified network analysis, latent profile invariance testing, and gender interaction analysis were not conducted; therefore, the findings should not be interpreted as gender-invariant. Fifth, some measures showed psychometric limitations in this sample. The factor structures of the SDS and BFI-2-SF were not fully supported, and several BFI-2-SF dimensions showed relatively low reliability. Thus, findings related to depressive subdimensions and personality traits should be interpreted cautiously and further validated in future studies.

## 5. Conclusions

This study examined the associations among depressive symptoms, physical activity, personality traits, and goal orientation in college students using network analysis, latent profile analysis, and multiple linear regression. The findings suggest that depressive symptoms were statistically connected with personality, motivational, and behavioral factors within a multidimensional association structure. In the overall network, psychological equivalents showed the highest strength centrality, while neuroticism showed prominent positive bridge expected influence between personality traits and depression-related indicators. In the regression models, task orientation, agreeableness, conscientiousness, and openness were negatively associated with depressive symptoms to varying degrees. Physical activity indicators showed limited direct associations with depressive symptoms, although walking was negatively associated with depressive symptoms in the maladaptive, low-motivation, inactive profile. Overall, these findings provide associative, network- and profile-based evidence for understanding depressive symptoms among college students. Given the cross-sectional design, these findings should be regarded as associative and hypothesis-generating. They may inform future longitudinal and intervention studies but should not be interpreted as causal evidence for specific intervention targets.

## Figures and Tables

**Table 1:** Sample Demographics by Grade and Gender.

Grade	Male	Female	Total	Proportion of Total	Proportion of Grade (Male)	Proportion of Grade (Female)
Freshman	73	260	333	36.55%	21.92%	78.08%
Sophomore	96	229	325	35.67%	29.54%	70.46%
Junior	50	162	212	23.27%	23.58%	76.42%
Senior	17	24	41	4.50%	41.46%	58.54%
Total	236	675	911	100.00%	25.91%	74.09%

**Table 2:** Descriptive Statistics of Study Variables.

Variable	*N*	Minimum	Maximum	Mean	Std. Deviation	Skewness	Kurtosis
MVPA	911	0	22,400	1089.5148	2046.73795	5.815	47.286
walking	911	0	13,860	710.5357	1016.90548	4.994	42.24
sedentary time	911	0	6000	241.4863	307.35929	10.367	165.51
extraversion	911	0	15	8.7278	1.90733	−0.293	0.351
agreeableness	911	3	15	10.9232	1.76928	−0.103	0
conscientiousness	911	5	15	9.8793	1.73924	0.2	0.028
neuroticism	911	3	15	8.8617	2.07777	−0.063	0.088
openness	911	3	15	10.0505	1.77519	0.1	0.551
self-orientation	911	6	30	21.2975	4.53368	−0.284	0.744
task-orientation	911	7	35	25.1416	5.10771	−0.554	1.261
pervasive affect	911	2	8	4.5071	1.15106	−0.098	0.142
physiological equivalents	911	8	26	16.202	3.57569	−0.098	−0.21
psychological equivalents	911	10	35	21.1965	4.37548	−0.171	0.195
depression total score	911	20	65	41.9056	7.68471	−0.369	−0.052
MVPA_Log	911	0	4.35	2.1774	1.31657	−0.832	−0.869
walking_Log	911	0	4.14	2.5483	0.58409	−1.021	2.934
sedentary_Log	911	0	3.78	2.1907	0.47771	−1.494	4.752

Note. MVPA = Moderate-to-Vigorous Physical Activity.

**Table 3:** Standardized coefficients (Z-scores).

Node	Strength	Betweenness	Closeness	Expected Influence
MVPA	−0.68	0.33	−1.22	0.18
walking	−0.82	−0.88	−1.6	0.01
sedentary time	−1.89	−1.22	−2	−1.14
extraversion	−0.31	−0.36	−0.07	−0.76
agreeableness	−0.25	0.33	0.63	0.12
conscientiousness	0.04	−0.53	0.33	−1.75
neuroticism	0.27	1.02	1.11	−0.8
openness	−0.46	0.85	0.35	0.3
self-orientation	0.47	0.16	0.33	1.97
task-orientation	1.43	0.85	0.46	1.47
pervasive affect	0.3	−1.22	0.19	0.19
physiological equivalents	−0.2	−1.22	0.19	0.43
psychological equivalents	2.1	1.89	1.3	−0.23

**Table 4:** Fit indices for latent profile models with one to six classes.

Class	Loglikelihood	AIC	BIC	Sample-Size Adjusted BIC	Entropy	*p*-Value
1	−17,672.587	35,385.174	35,481.464	35,417.947	-	-
2	−17,087.096	34,236.192	34,385.443	34,286.991	0.995	<0.001
3	−16,831.13	33,746.26	33,948.471	33,815.085	0.858	<0.001
4	−16,700.279	33,506.558	33,761.729	33,593.408	0.873	0.6715
5	−16,593.928	33,315.856	33,623.986	33,420.731	0.851	0.056
6	−16,480.111	33,110.222	33,471.313	33,233.123	0.91	0.4136

**Table 5:** Predictors of depressive symptoms: Total sample and three latent profiles.

Variable	Total Sample	Class 1	Class 2	Class 3
Model fit indices				
Adjusted R^2^	0.389	0.318	0.197	0.348
F	58.901 ***	9.906 ***	13.106 ***	13.034 ***
Standardized coefficients (β)				
extraversion	0.011	−0.032	0.014	0.073
agreeableness	−0.118 ***	−0.145 *	−0.118 **	−0.099
conscientiousness	−0.137 ***	−0.211 **	−0.072	−0.126 *
neuroticism	0.366 ***	0.396 ***	0.326 ***	0.469 ***
openness	−0.089 **	−0.029	−0.177 ***	0.041
self-orientation	0.051	0.119	0.139 *	−0.138
task-orientation	−0.252 ***	−0.271 **	−0.312 ***	−0.091
MVPA	−0.008	−0.008	−0.045	−0.032
walking	−0.043	−0.028	0.005	−0.132 *
sedentary time	−0.011	−0.118	−0.013	0.02

Note. Values are standardized regression coefficients. Significance levels: * *p* < 0.05, ** *p* < 0.01, *** *p* < 0.001. MVPA = moderate-to-vigorous physical activity.

## Data Availability

The data used in this study are available from the corresponding author upon request.

## References

[B1-behavsci-16-01206] Aguirre P., Michelini Y., Bravo A. J., Pautassi R. M., Pilatti A. (2024). Association between personality traits and symptoms of depression and anxiety via emotional regulation and distress tolerance. PLoS ONE.

[B2-behavsci-16-01206] Ainsworth B. E., Haskell W. L., Herrmann S. D., Meckes N., Bassett D. R., Tudor-Locke C., Greer J. L., Vezina J., Whitt-Glover M. C., Leon A. S. (2011). 2011 Compendium of physical activities: A second update of codes and MET values. Medicine & Science in Sports & Exercise.

[B3-behavsci-16-01206] Borghuis J., Bleidorn W., Sijtsma K., Branje S., Meeus W. H., Denissen J. J. A. (2020). Longitudinal associations between trait neuroticism and negative daily experiences in adolescence. Journal of Personality and Social Psychology.

[B4-behavsci-16-01206] Chekroud S. R., Gueorguieva R., Zheutlin A. B., Paulus M., Krumholz H. M., Krystal J. H., Chekroud A. M. (2018). Association between physical exercise and mental health in 1.2 million individuals in the USA between 2011 and 2015: A cross-sectional study. The Lancet Psychiatry.

[B5-behavsci-16-01206] Cheng G., Qiu W., Nie Y., Ma W., Wang X., Wang H., Cui H., Liu C. (2026). Exploring the associations of physical activity, social support, and psychological resilience in college students: A network analysis. International Journal of Mental Health Promotion.

[B6-behavsci-16-01206] Cohen J., Cohen P., West S. G., Aiken L. S. (2013). Applied multiple regression/correlation analysis for the behavioral sciences.

[B7-behavsci-16-01206] Collins L. M., Lanza S. T. (2013). Latent class and latent transition analysis: With applications in the social, behavioral, and health sciences.

[B8-behavsci-16-01206] Craig C. L., Marshall A. L., Sjöström M., Bauman A. E., Booth M. L., Ainsworth B. E., Pratt M., Ekelund U., Yngve A., Sallis J. F., Oja P. (2003). International physical activity questionnaire: 12-country reliability and validity. Medicine & Science in Sports & Exercise.

[B9-behavsci-16-01206] Dagsdóttir B. E., Kristjánsdóttir H., Vésteinsdóttir V., Thorsdottir F. (2023). Task and ego orientation in sport questionnaire: A Mokken scale analysis. SAGE Open.

[B10-behavsci-16-01206] Deng Q., Zheng B., Chen J. (2020). The relationship between personality traits, resilience, school support, and creative teaching in higher school physical education teachers. Frontiers in Psychology.

[B11-behavsci-16-01206] Dong S., Ge H., Su W., Guan W., Li X., Liu Y., Yu Q., Qi Y., Zhang H., Ma G. (2024). Enhancing psychological well-being in college students: The mediating role of perceived social support and resilience in coping styles. BMC Psychology.

[B12-behavsci-16-01206] Duda J. L., Nicholls J. G. (1992). Dimensions of achievement motivation in schoolwork and sport. Journal of Educational Psychology.

[B13-behavsci-16-01206] Epskamp S., Borsboom D., Fried E. I. (2018). Estimating psychological networks and their accuracy: A tutorial paper. Behavior Research Methods.

[B14-behavsci-16-01206] Foygel R., Drton M. (2010). Extended Bayesian information criteria for Gaussian graphical models. Advances in Neural Information Processing Systems.

[B15-behavsci-16-01206] IPAQ Research Committee (2005). Guidelines for data processing and analysis of the International Physical Activity Questionnaire (IPAQ)-short and long forms.

[B16-behavsci-16-01206] Jiang B. C., Zhang J., Yang M., Yang H. D., Zhang X. B. (2025). Prevalence and risk factors of depressive and anxiety symptoms and functional constipation among university students in Eastern China. World Journal of Psychiatry.

[B17-behavsci-16-01206] Jones P. J., Ma R., McNally R. J. (2021). Bridge centrality: A network approach to understanding comorbidity. Multivariate Behavioral Research.

[B18-behavsci-16-01206] Li S., Wang X., Wang P., Qiu S., Xin X., Wang J., Zhao J., Zhou X. (2023). Correlation of exercise participation, behavioral inhibition and activation systems, and depressive symptoms in college students. Scientific Reports.

[B19-behavsci-16-01206] Liu C., Ma W., Qiu W., Qu G., Gao R. (2026). Network structure and latent profile analysis of adolescent sports moral character, goal orientations, and self-efficacy. International Journal of Sport and Exercise Psychology.

[B20-behavsci-16-01206] Lubans D., Richards J., Hillman C., Faulkner G., Beauchamp M., Nilsson M., Kelly P., Smith J., Raine L., Biddle S. (2016). Physical activity for cognitive and mental health in youth: A systematic review of mechanisms. Pediatrics.

[B21-behavsci-16-01206] Ma D., Akram H., Li S. (2026). Assessing the role of physical activity in shaping students’ academic motivation: The mediating role of mental health. BMC Public Health.

[B22-behavsci-16-01206] Ma W., Wang X., Nie Y., Gao R., Liu C. (2025). Association between neuroticism and physical activity: A systematic review and meta-analysis. Frontiers in Human Neuroscience.

[B23-behavsci-16-01206] Meiering M. S., Weigner D., Gruzman R., Enge S., Grimm S. (2025). An investigation of the interaction of trait repetitive negative thinking and neuroticism on brain activity during negative self-referential processing: A cross-sectional fMRI study. Social Cognitive and Affective Neuroscience.

[B24-behavsci-16-01206] Meinshausen N., Bühlmann P. (2006). High-dimensional graphs and variable selection with the lasso. The Annals of Statistics.

[B25-behavsci-16-01206] Mikkelsen A. T., Jensen K. H. R., Jørgensen M. B., Frokjaer V. G., Dam V. H. (2024). No association between serotonin 4 receptor brain binding and personality trait neuroticism—A positron emission tomography study in depressed patients and healthy individuals. Neuroscience Applied.

[B26-behavsci-16-01206] Miscioscia M., Poli M., Gubello A., Simonelli A., Gatta M., Gato J., Rigo P. (2022). Influence of the COVID-19 pandemic on Italian LGBT+ young adults’ mental health: The role of neuroticism and family climate. International Journal of Environmental Research and Public Health.

[B27-behavsci-16-01206] Nie Y., Wang T., Guo M., Zhou F., Ma W., Qiu W., Gao J., Liu C. (2025a). The relationship between physical activity, life satisfaction, emotional regulation, and physical self-esteem among college students. Scientific Reports.

[B28-behavsci-16-01206] Nie Y., Wang W., Liu C., Wang T., Zhou F., Gao J. (2025b). Social challenges on university campuses: How does physical activity affect social anxiety? The dual roles of loneliness and gender. Behavioral Sciences.

[B29-behavsci-16-01206] Peng C., Chen J., Wu H., Liu Y., Liao Y., Wu Y., Zheng X. (2021). Father-child conflict and Chinese adolescent depression: A moderated mediation model. Frontiers in Psychology.

[B30-behavsci-16-01206] Pengpid S., Peltzer K. (2019). High sedentary behaviour and low physical activity are associated with anxiety and depression in Myanmar and Vietnam. International Journal of Environmental Research and Public Health.

[B31-behavsci-16-01206] Prochnow T., Delgado H., Patterson M. S., Umstattd Meyer M. R. (2020). Social network analysis in child and adolescent physical activity research: A systematic literature review. Journal of Physical Activity and Health.

[B32-behavsci-16-01206] Qiu W., Huang C., Xiao H., Nie Y., Ma W., Zhou F., Liu C. (2025). The correlation between physical activity and psychological resilience in young students: A systematic review and meta-analysis. Frontiers in Psychology.

[B33-behavsci-16-01206] Qu G., Jia F., Liu J., Wang X., Tang G., Gu Z., Nie Y., Liu C. (2026). The relationships among exercise participation, self-compassion and academic stress in classroom contexts: Based on latent profiles and mediation analyses. International Journal of Mental Health Promotion.

[B34-behavsci-16-01206] R Core Team (2016). R: A language and environment for statistical computing.

[B35-behavsci-16-01206] Robertson R., Robertson A., Jepson R., Maxwell M. (2012). Walking for depression or depressive symptoms: A systematic review and meta-analysis. Mental Health and Physical Activity.

[B36-behavsci-16-01206] Soto C. J., John O. P. (2017). Short and extra-short forms of the Big Five Inventory-2: The BFI-2-S and BFI-2-XS. Journal of Research in Personality.

[B37-behavsci-16-01206] Tang D. D., Wen Z. L. (2020). Common method bias testing: Problems and suggestions. Psychological Science.

[B38-behavsci-16-01206] Taylor S., Martin J., Wilson O. W., Elliot L., Bopp M. (2025). The impact of physical activity enjoyment, exercise self-efficacy, recording physical activity, and exercise goal setting on physical activity levels of college students. Recreational Sports Journal.

[B39-behavsci-16-01206] Truninger M., Fernández-i-Marín X., Batista-Foguet J. M., Boyatzis R. E., Serlavós R. (2018). The power of EI competencies over intelligence and individual performance: A task-dependent model. Frontiers in Psychology.

[B40-behavsci-16-01206] Wang W., Song S., Chen X., Yuan W. (2021). When learning goal orientation leads to learning from failure: The roles of negative emotion coping orientation and positive grieving. Frontiers in Psychology.

[B41-behavsci-16-01206] Wang X. Y., Wang Z. W., Jiang D. L., Liu C., Xing W. Y., Yuan Z. T., Cui L. B., Wu S. J., Ren L. (2025). Personality perspective on depression and anxiety symptoms among Chinese adolescents and young adults: A two-sample network analysis. BMC Psychiatry.

[B42-behavsci-16-01206] Wang Y., Li Y. (2022). Physical activity and mental health in sports university students during the COVID-19 school confinement in Shanghai. Frontiers in Public Health.

[B43-behavsci-16-01206] Woo S. E., Jebb A. T., Tay L., Parrigon S. (2018). Putting the person in the center: Review and synthesis of person-centered approaches and methods in organizational science. Organizational Research Methods.

[B44-behavsci-16-01206] World Health Organization (2022). World mental health report: Transforming mental health for all.

[B45-behavsci-16-01206] Zhang M., Pan J., Shi W., Qin Y., Guo B. (2024). The more self-control, the more anxious? A network analysis study of the relationship between self-control and psychological anxiety among Chinese university students. BMC Psychology.

[B46-behavsci-16-01206] Zhang X. H., Miao H., Yan W. J., Zheng T. T., Lyu H. Z. (2026). Multilayer network analysis of mental health symptoms in UK university students: Association patterns of depression, loneliness, and suicidal ideation. Frontiers in Psychiatry.

[B47-behavsci-16-01206] Zhang Y. (2025). The impact of Big Five personality traits on exercise motivation and mental health in college students: The mediating role of emotional intelligence. Acta Psychologica.

[B48-behavsci-16-01206] Zhao B., Deng X., Zhou Z. (2025). Connection between college students’ sports activities, depression, and anxiety: The mediating role of self-esteem. BMC Psychology.

[B49-behavsci-16-01206] Zhou H., Dai X., Lou L., Zhou C., Zhang W. (2021). Association of sedentary behavior and physical activity with depression in sport university students. International Journal of Environmental Research and Public Health.

[B50-behavsci-16-01206] Zhu J. H., Li S. F., Wang P., Xin X., Zhao Q., Chen S. C., Wang X. (2023). Correlation and pathways of behavioral activation systems mediating physical activity level and depressive symptoms among college students. World Journal of Psychiatry.

[B51-behavsci-16-01206] Zung W. W. (1965). A self-rating depression scale. Archives of General Psychiatry.

